# Correction: Risk of hepatitis B virus reactivation in the treatment of HBsAg and HBV DNA double-negative lymphoma patients

**DOI:** 10.1371/journal.pone.0356789

**Published:** 2026-08-20

**Authors:** The PLOS One Staff

In Table 1, a percentage sign (%) is already displayed in the header (the first row of the table), and there is a redundant “%” in the newly revised data for duration of treatment. Please see the correct Table 1 here.

In the Results subsection of the Abstract, there is an error in the last sentence of the paragraph. The correct sentence is: Conversely, no statistically significant differences in HBV reactivation rates were observed between patients of different genders (P = 0.637, OR = 0.855) or across varying treatment durations (P = 0.837).

In the Impact of treatment duration on HBV reactivation subsection of the Results, there are errors in the paragraph. The correct paragraph is: Since patients with a treatment duration exceeding 30 months in each group are fewer than 20, they are excluded from the intergroup comparison of treatment durations. And there was no statistically significant difference in the incidence of HBV reactivation among lymphoma patients who underwent different treatment durations (P = 0.837). Except for patients who did not experience HBV reactivation within the 24–27 months treatment period, HBV reactivation occurred in all other stages. Among those who experienced reactivation, the highest frequency of HBV reactivation (3.41%) was observed during the 15–18 months treatment period, although there were only 3 cases. In contrast, 14 cases (2.71%) of HBV reactivation occurred during the 3–6 months treatment period (Table 1).

The publisher apologizes for the errors.

**Table 1:** The results of different factors affecting HBV reactivation.

Factors	Classifications	The number of observations	HBV reactivation		P-value
Yes (%)	No (%)
HBcAb	Positive	410	41 (10.00)	369 (90.00)	<0.001
	Negative	206	3 (1.46)	203 (98.54)	
Age (Y)	0<Y≤20	18	0 (0.00)	18 (100.00)	0.002
	21<Y≤30	51	0 (0.00)	51 (100.00)	
	31<Y≤40	57	0 (0.00)	57 (100.00)	
	41<Y≤50	93	4 (4.30)	89 (95.70)	
	51<Y≤60	157	11 (7.01)	146 (92.99)	
	61<Y≤70	147	17 (11.56)	130 (88.44)	
	>70	93	12 (12.90)	81 (87.10)	
Gender	Male	358	24 (6.70)	334 (93.30)	0.637
	Female	258	20 (7.75)	238 (92.25)	
Duration of treatment (M)	0<M≤3	616	12 (1.95)	604 (98.05)	0.837
	3<M≤6	517	14 (2.71)	503(97.29)	
	6<M≤9	297	5 (1.68)	292 (98.32)	
	9<M≤12	173	2 (1.16)	171 (98.84)	
	12<M≤15	115	1 (0.87)	114 (99.13)	
	15<M≤18	88	3 (3.41)	85 (96.59)	
	18<M≤21	69	1 (1.45)	68 (98.55)	
	21<M≤24	56	1 (1.79)	55 (98.21)	
	24<M≤27	46	0 (0.00)	46 (100.00)	
	27<M≤30	31	1 (3.23)	30 (96.77)	
	30<M≤33	18	0 (0.00)	18 (100.00)	
	33<M≤36	8	2 (25.00)	6 (75.00)	
	36<M≤39	5	1 (20.00)	4 (80.00)	
	39<M≤42	4	1 (25.00)	3 (75.00)	
	42<M≤45	3	0 (0.00)	3 (100.00)	
	45<M≤48	2	0 (0.00)	2 (100.00)	

For HBcAb status, the OR of HBV reactivation in HBcAb (+) individuals relative to HBcAb (-) individuals is 7.52, with a 95% CI of 2.24 to 25.22. For gender factor, the OR is 0.855, with a 95% CI of 0.455 to 1.604. Among the 616 patients, the age range is from 14 to 88 years, with an average age of 53.9 years. M: Months, Y: Years.
