# Supplementary material for: Risk of hepatitis B virus reactivation in the treatment of HBsAg and HBV DNA double-negative lymphoma patients
Source: PLoS One. 2025 Sep 17;20(9):e0332421. doi: 10.1371/journal.pone.0332421 (PMC12443279; doi:10.1371/journal.pone.0332421)
Supplement: Attachment — Submitted filename: Response to Reviewers.docx [file pone.0332421.s003.docx]

To reviewer #1

Thank you for your insightful comments! We truly appreciate your recognition of our study's interesting idea and standard methodology.

To reviewer #2

Thank you so much for your constructive and valuable comments! We're delighted that you recognized the well-designed nature of our study and its contributions to HBV reactivation management. Here are our point-by-point responses:

1. We have added brief demographic information of participants to the abstract as suggested.
2. The discussion section has been expanded to better discuss the results from virological aspects by comparing them with other similar studies.
3. We have mentioned HBV treatment options in China in the Discussion section.
4. We have deeply recognized the limitations of this study and mentioned them in the discussion section, which provides a fundamental direction for our further in-depth research.

Your feedback has been incredibly helpful in improving our manuscript. Thanks again!

To reviewer #3

Thank you for your insightful and valuable comments! We're grateful for your recognition of the significance of our study and its new insights. Below are our point-by-point responses to your comments:

Reply to the major comments:

1. We have revised the introduction section to fully emphasize the necessity of studying the risk of HBV reactivation in HBsAg and HBV DNA double-negative patients, and to highlight the value of this study.
2. We have revised the study population section of the manuscript to include detailed explanations of patient inclusion and exclusion criteria.
3. We have added an explanation of the frequency of patient testing for HBsAg, HBcAb, and HBV DNA in the detection methods section of the manuscript.

4. Indeed, during the course of our research, we encountered certain objective limitations, including incomplete patient data records, as well as constraints on study time and resources. Owing to these factors, we were unable to incorporate some widely acknowledged predictive factors into our analysis. This undoubtedly detracted from the overall comprehensiveness of our risk assessment, and for this, we offer our sincere apologies.

Nevertheless, our study possesses its own distinct value. It specifically targets a subset of lymphoma patients who test negative for both HBsAg and HBV DNA prior to treatment—a population that has been relatively understudied in terms of risk factors for HBV reactivation. Our findings reveal that HBV reactivation can still occur in patients even when initial tests for HBsAg and HBV DNA are negative. Moreover, we observed cases of HBV reactivation in patients who were HBcAb negative. These insights provide crucial cues for clinicians to focus on this often-overlooked patient group and underscore the necessity of close monitoring for them.

5. This study focuses on a specific population of lymphoma patients who are both HBsAg and HBV DNA negative before treatment, with the aim of exploring potential risk factors that may affect HBV reactivation. The chi-square test and Fisher's exact test can visually present the association between various factors (such as HBcAb status, age group, gender, treatment duration) and HBV reactivation, providing a basic direction for further in-depth research. Due to the retrospective analysis of this study, there are certain difficulties in collecting data on some potential confounding factors, such as incomplete detailed information on patients' lifestyles, underlying diseases, etc. This makes the adjustment of confounding factors in multivariate analysis not accurate and comprehensive enough, which may lead to biased results. Therefore, we chose to first screen for possible risk factors through univariate analysis, laying the foundation for future prospective studies and conducting in-depth analysis using multivariate statistical methods.

6. During the statistical analysis phase of this study, we have fully considered the impact of treatment duration and sample size on the reliability of the results. In fact, the number of enrolled cases in each group with a treatment duration exceeding 30 months is less than 20. Considering that a small sample size may lead to unstable and biased statistical results, which cannot accurately reflect the true situation, we have not included the data of treatment duration greater than 30 months in the formal inter group comparative analysis. In the manuscript, we actually briefly mentioned this processing method, which may have been overlooked by you due to the lack of prominent expression. We deeply apologize for this. To address this, we have refined the wording in the main text to ensure that this crucial information is conveyed more clearly and explicitly.
7. From the perspective of research design, this study aims to focus on the risk factors for serum HBV reactivation during treatment in patients with HBsAg and HBV DNA double-negative lymphoma before treatment. In the context of limited resources, in order to ensure the feasibility of the study and the achievement of core objectives, we chose to prioritize the collection and analysis of data directly related to serological HBV reactivation, and did not include the collection of data on liver function and severity of hepatitis attacks in this study.

Although serological reactivation is not always associated with clinically significant liver events, identifying risk factors for serological HBV reactivation remains important for clinical practice. It can help clinical doctors identify high-risk patients who may experience HBV reactivation in advance, and take corresponding preventive measures, such as strengthening monitoring, to reduce the adverse effects of HBV reactivation on patients to a certain extent.

8. We have deeply recognized the limitations of this study and mentioned them in the discussion section, which provides a fundamental direction for our further in-depth research.
9. We have revised the discussion section, delving deeper into the reasons behind the higher probabilities of HBV reactivation in HBcAb positive patients and the elderly by analyzing the mechanisms of HBV reactivation.

Reply to the minor comments:

1. Thank you very much for your suggestions. We have revised the descriptions of the positive criteria for HBsAg, HBcAb, and HBV DNA detection methods in the manuscript.

Your feedback has been instrumental in improving the quality and applicability of our study. Thank you again for your time and expertise!
