# Supplementary material for: Risk of hepatitis B virus reactivation in the treatment of HBsAg and HBV DNA double-negative lymphoma patients
Source: PLoS One. 2025 Sep 17;20(9):e0332421. doi: 10.1371/journal.pone.0332421 (PMC12443279; doi:10.1371/journal.pone.0332421)
Supplement: Attachment — Submitted filename: Response_to_Reviewers_auresp_2.docx [file pone.0332421.s004.docx]

To reviewer #2

1. Thank you for your constructive feedback regarding the inclusion of odds ratios (ORs) in Table 1. As suggested, we have calculated and added the ORs along with their corresponding 95% confidence intervals (CIs) to quantify the strength of association between HBcAb status, gender, and HBV reactivation.
2. Thank you for your insightful comment. We acknowledge the possibility of nosocomial infection as an alternative explanation for the positive HBV cases observed during treatment. To address this, we will add a section in the discussion to clarify that while nosocomial infection cannot be entirely ruled out, our strict diagnostic criteria for HBV reactivation and the infection control measures in place at our hospital support the interpretation of these cases as reactivation events.

Your feedback has been incredibly helpful in improving our manuscript. Thanks again!

To reviewer #3

Thank you for your insightful and valuable comments! We're grateful for your recognition of the significance of our study and its new insights. Below are our point-by-point responses to your comments:

1. The issues you raised are indeed critical and hold significant importance for enhancing the scientific rigor and credibility of our study. We have engaged in thorough deliberation regarding the three key points you identified, and have subsequently revised and improved the Discussion section accordingly.
2. We fully appreciate the significance of incorporating variables such as past antiviral treatment history, comorbidities, and details of immunosuppressive regimens (e.g., the use of rituximab or corticosteroids) in assessing the risk of HBV reactivation. However, based on the core scientific question of this study (the risk of HBV reactivation in lymphoma patients with both HBsAg and HBV DNA negativity), after careful deliberation by the research team, these variables were not included in the core analysis model for the following main reasons:

- This study aims to address a critical knowledge gap in current clinical practice—whether lymphoma patients with both HBsAg and HBV DNA negativity still face a risk of HBV reactivation. Due to the absence of positive evidence for traditional serological markers (HBsAg) and viral load (HBV DNA), such patients are often incorrectly classified as having "inactive infection" and thus do not receive prophylactic antiviral therapy.
- Despite the exclusion of the aforementioned variables, we minimized heterogeneity from the outset and ensured the reliability of our results by implementing stringent inclusion criteria: selecting only patients with both HBsAg and HBV DNA negativity who had not received prophylactic antiviral therapy.

We fully concur with the reviewer's viewpoint on the completeness of variables and plan to explore the following issues in subsequent studies:

- Validate our current findings in a larger cohort of double-negative patients and incorporate variables such as antiviral treatment history and comorbidities for multivariable adjustment.
- Conduct mechanistic studies (e.g., detection of cccDNA in liver tissue) to elucidate the molecular biological basis of occult HBV reactivation.

We have refined Figure 1 to clearly indicate the specific number of patients excluded at each step.

3. Regarding the frequency of patient visits, it is indeed tailored to each patient's specific treatment plan, rather than adhering to a fixed, uniform schedule such as every 1 - 2 weeks or monthly. This personalized approach is adopted to optimize treatment outcomes based on the specific characteristics and needs of each patient's condition. However, it is important to emphasize that we ensure HBsAg, HBcAb, and HBV DNA tests are conducted for each patient during every treatment session, thereby guaranteeing the reliability of the timing of HBV reactivation occurrence in individual cases.

As for the handling of missing data, we would like to clarify that patients who did not undergo regular testing as per the treatment plan were excluded during the initial screening based on inclusion criteria. The implementation of this stringent standard aims to ensure the reliability of the HBV reactivation detection timeline and guarantee the comparability of follow-up situations among different subgroups in our analysis. We hope these additional details can fully address your queries. We are committed to providing clear and comprehensive information to support the validity and reliability of our study findings.

4. This study focuses on filling a critical knowledge gap in current clinical practice, namely, whether lymphoma patients with both HBsAg and HBV DNA negativity still face the risk of HBV reactivation. Due to the lack of positive evidence for traditional serological markers (HBsAg) and viral load (HBV DNA), such patients are often misjudged as having "inactive infection" and thus do not receive prophylactic antiviral therapy. Therefore, our primary objective is to minimize heterogeneity from the outset by employing strict inclusion criteria, selecting only patients who are negative for both HBsAg and HBV DNA and have not received prophylactic antiviral therapy. This ensures that our study can precisely focus on particular group and clarify whether there is a risk of HBV reactivation.

Although we understand that factors such as chemotherapy regimens, corticosteroid use, baseline liver function, and HBsAb titers have significant impacts on HBV reactivation, incorporating these variables into the analysis would substantially increase the complexity and heterogeneity of the study. Different chemotherapy regimens vary in their mechanisms of action, dosage intensities, and treatment cycles; corticosteroid use also differs in terms of dosage, duration, and mode of administration; baseline liver function indicators are influenced by multiple factors, and inconsistent results may arise from different detection methods and standards; moreover, the dynamic changes in HBsAb titers are complex. If all these factors were included in the core analysis model, it might disperse the research focus and make it difficult to accurately assess the impact of the core factor—HBsAg and HBV DNA double negativity—on the risk of HBV reactivation.

Nevertheless, we fully agree with your viewpoint regarding the completeness of variables. Although this study did not provide descriptive statistics or stratified analyses to cover these well - recognized risk factors, we plan to carry out the following work in subsequent studies to make up for this deficiency:

- Expand the sample size and incorporate more variables: Validate the current findings in a larger cohort of double - negative patients and meticulously record information such as chemotherapy regimens, corticosteroid use, baseline liver function indicators, and anti - HBs titers. Conduct multivariate adjusted analyses to comprehensively evaluate the impacts of these factors on the risk of HBV reactivation.
- Delve deeper into the underlying mechanisms: Conduct mechanistic research, such as detecting cccDNA in liver tissues, to further clarify the molecular biological basis of occult HBV reactivation and how chemotherapy regimens, corticosteroid use, and other factors promote HBV reactivation by influencing virus - host interactions.

5. Firstly, the core objective of this study is to conduct a preliminary exploration of the risk of HBV reactivation in double-negative lymphoma patients. We aim to swiftly obtain basic information regarding the relationships between the two variables, HBcAb (+) and advanced age, and HBV reactivation through simple and straightforward univariate analysis. This will provide direction and preliminary evidence for subsequent more in-depth research. Univariate analysis can present data in a concise and clear manner, enabling readers to quickly grasp the basic associations between each factor and the outcome variable.

Secondly, the data in this study are collected retrospectively, which entails certain limitations in terms of data completeness and accuracy. Records of some potential confounding factors, such as treatment types and comorbidities, may be missing or inaccurate. If we forcibly incorporate these factors into a multivariate model, the model may become unstable due to data quality issues, leading to unreliable conclusions. We are concerned that such inaccurate model results will not only fail to offer valuable references for clinical practice but may also mislead clinical decision - making.

Furthermore, as an exploratory study, this research has a relatively limited sample size. When incorporating too many covariates into multivariate modeling with a limited sample size, overfitting is likely to occur. This means that the model may perform well on the sample data but have poor generalization ability in practical applications, failing to accurately predict new data scenarios. We hope to fully consider the application of multivariate modeling in subsequent studies with larger sample sizes to ensure the reliability and practicality of the research results.

We fully acknowledge that, as you have pointed out, this is a major methodological limitation affecting the interpretability of our study's conclusions. In the discussion section of the paper, we have elaborated on this limitation and reminded readers to carefully consider the influence of potential confounding factors when interpreting the research results. Meanwhile, we have also clearly stated in the research prospects that we will carry out larger - scale and more rigorously designed studies in the future, attempting to use multivariate models to further delve into the relationships between HBcAb (+), advanced age, and other potential factors and HBV reactivation, with the aim of providing more comprehensive and accurate evidence for clinical practice.

6. The core positioning of this study is to conduct a preliminary exploration of the risk of serological HBV reactivation in patients with double-negative lymphoma. Our research focus is on using serological indicators to initially screen and understand the basic situation of HBV reactivation, thereby laying a foundation for subsequent more in-depth and comprehensive research. In this preliminary exploration stage, we mainly concentrate on changes at the serological level, aiming to rapidly obtain basic information regarding the relationships between variables such as HBcAb (+) and advanced age and serological HBV reactivation.

Regarding your suggestion to obtain ALT or clinical hepatitis data to reveal clinical reactivation, we are well aware that this is of paramount importance for a comprehensive assessment of patients' conditions and the actual impact of HBV reactivation. However, as this is a preliminary exploratory study and the data are collected retrospectively, the records in the medical record system exhibit a certain degree of dispersion and redundancy. For instance, in the case of ALT data, patients may have undergone multiple tests during a single treatment period, which poses significant challenges to the unified collection and organization of the data.

We have already elaborated on this limitation in the discussion section of the paper, reminding readers to carefully consider the potential impacts of the lack of clinical reactivation data when interpreting the research results. Meanwhile, we have also clearly stated in the research prospects that we will carry out larger-scale and more rigorously designed studies in the future, further improve the data collection system, and incorporate clinical reactivation data such as ALT and clinical hepatitis to more comprehensively and accurately evaluate HBV reactivation, thus providing more valuable references for clinical practice.

Your feedback has been instrumental in improving the quality and applicability of our study. Thank you again for your time and expertise!
